# Mechanical loading stimulates hypertrophy in tissue‐engineered skeletal muscle: Molecular and phenotypic responses

**DOI:** 10.1002/jcp.28923

**Published:** 2019-06-10

**Authors:** Kathryn W. Aguilar‐Agon, Andrew J. Capel, Neil R.W. Martin, Darren J. Player, Mark P. Lewis

**Affiliations:** ^1^ School of Sport, Exercise and Health Sciences Loughborough University Loughborough United Kingdom; ^2^ Division of Surgery University College London London United Kingdom

**Keywords:** hypertrophy, mTORC1, myotubes, skeletal muscle, tissue engineering

## Abstract

Mechanical loading of skeletal muscle results in molecular and phenotypic adaptations typified by enhanced muscle size. Studies on humans are limited by the need for repeated sampling, and studies on animals have methodological and ethical limitations. In this investigation, three‐dimensional skeletal muscle was tissue‐engineered utilizing the murine cell line C2C12, which bears resemblance to native tissue and benefits from the advantages of conventional in vitro experiments. The work aimed to determine if mechanical loading induced an anabolic hypertrophic response, akin to that described in vivo after mechanical loading in the form of resistance exercise. Specifically, we temporally investigated candidate gene expression and Akt‐mechanistic target of rapamycin 1 signalling along with myotube growth and tissue function. Mechanical loading (construct length increase of 15%) significantly increased insulin‐like growth factor‐1 and MMP‐2 messenger RNA expression 21 hr after overload, and the levels of the atrophic gene MAFbx were significantly downregulated 45 hr after mechanical overload. In addition, p70S6 kinase and 4EBP‐1 phosphorylation were upregulated immediately after mechanical overload. Maximal contractile force was augmented 45 hr after load with a 265% increase in force, alongside significant hypertrophy of the myotubes within the engineered muscle. Overall, mechanical loading of tissue‐engineered skeletal muscle induced hypertrophy and improved force production.

## 1 INTRODUCTION

Skeletal muscle exhibits a high degree of plasticity and is responsive to both increased (exercise) and decreased (disuse) mechanical loading (Bodine, 2013; Sandri, 2008). In particular, mechanical loading greatly influences skeletal muscle size and its ability to produce force, as well as muscle cell differentiation and matrix remodelling (Lo Presti, Hopps, & Caimi, 2017). Indeed, in rodents, chronic resistance training (Mikesky, Giddings, Matthews, & Gonyea, 1991) has been consistently shown to enhance muscle mass, fibre size, and muscle function. Similarly, in humans, 6–24 weeks of resistance exercise has been shown to induce a large increase in the muscle fibre area and maximal force production (Adams, Hather, Baldwin, & Dudley, 1993; Dons, Bollerup, Bonde‐Petersen, & Hancke, 1979; Liu, Schlumberger, Wirth, Schmidtbleicher, & Steinacker, 2003; MacDougall, Sale, Elder, & Sutton, 1982). Skeletal muscle size and function are strongly associated with morbidity, mortality (Shibahashi, Sugiyama, Kashiura, & Hamabe, 2017), and quality of life (Carmeli, Coleman, & Reznick, 2002; Kortebein et al., 2008). Therefore, the determination of appropriate models to further our understanding of the plasticity of skeletal muscle to increased and/or decreased loading is critical to understanding muscle‐wasting disease.

Tissue engineered three‐dimensional (3D) skeletal muscle models have been developed in vitro from primary rodents (Smith, Passey, Greensmith, Mudera, & Lewis, 2012), cell lines (Player et al., 2014), and human muscle precursor cells (Martin et al., 2013; Mudera, Smith, Brady, & Lewis, 2010; Powell, Smiley, Mills, & Vandenburgh, 2002). Engineered muscles are fabricated by seeding cells on or within an extra cellular matrix (ECM) and placing them under uniaxial tension between two fixed points, allowing the differentiation of highly aligned myotubes capable of force generation, similar to that of native muscle tissue (Huang, Dennis, Larkin, & Baar, 2005; Kasper, Turner, Martin, & Sharples, 2018; Khodabukus, Paxton, Donnelly, & Baar, 2007; Martin et al., 2017). Common ECM materials include fibrin (Huang et al., 2005), type I collagen (Cheema et al., 2005; Cheema, Yang, Mudera, Goldspink, & Brown, 2003; Player et al., 2014; Smith et al., 2012) and laminin (Dennis & Kosnik, 2000), either alone or in combination with Matrigel^™^, which promotes cell growth and differentiation (Powell et al., 2002; Shansky, Del Tatto, Chromiak, & Vandenburgh, 1997). Because type I collagen is the most abundant of these matrices in skeletal muscle tissue (Kovanen, 2002), it is an ideal scaffold for engineering in‐vivo‐like native muscle tissue. Crucially, such systems enable the coupling of mechanistic molecular outputs with morphological and functional measures within a highly controlled environment, without the methodological and ethical constraints of repeated biopsy sampling or animal sacrifice. As such, tissue‐engineered 3D skeletal muscle provides an appropriate system to explore skeletal muscle adaptations to mechanical loading.

Loading of skeletal muscle in vitro was traditionally accomplished through mechanical stretch and was first established in monolayer cultures of avian myotubes (Vandenburgh & Kaufman, 1979; Vandenburgh, Hatfaludy, Karlisch, & Shansky, 1989). In this seminal work, cyclic mechanical stretch of collagen embedded monolayer myotubes resulted in an increase in protein synthesis and myotube diameters and changes in the characteristic of skeletal muscle hypertrophy in vivo. Furthermore, it has been established that mechanical loading of monolayer myotube cultures results in the activation of the mechanistic target of rapamycin (mTOR) signalling cascade (Sasai et al., 2010), which has now been well‐established for its importance in regulating both protein synthesis and cellular growth (Bodine et al., 2001; Rommel et al., 2001; Wullschleger, Loewith, & Hall, 2006) and is activated after mechanical loading in humans and animals (Bodine, 2006; Bodine et al., 2001; Goodman et al., 2011; Reynolds, Bodine, & Lawrence, 2002).

Mechanical loading of bioengineered muscle has most frequently been used as a method to condition skeletal muscle during myotube development, enabling enhanced orientation, density, and even sarcomeric assembly of myofibers (Boonen et al., 2010; Moon du, Christ, Stitzel, Atala, & Yoo, 2008; Okano & Matsuda, 1997), resulting in a model more greatly analogous to native skeletal muscle. Because it has been established that during myotube development, mechanical loading of tissue engineered 3D skeletal muscle potently induced insulin‐like growth factor‐1 (IGF‐1) messenger RNA (mRNA) expression (Cheema et al., 2005), mechanical loading of such systems may drive a hypertrophic response, given that IGF‐1 is implicated in skeletal muscle growth (Velloso, 2008). Whilst data surrounding mechanical loading in postmitotic myotube cultures in 3D are limited, our laboratory has previously demonstrated that both ramp and static loading over a 60 min period in collagen‐based tissue‐engineered 3D skeletal muscle seeded with C2C12 cells resulted in upregulated gene transcripts associated with muscle growth and remodelling such as IGF‐1 and matrix metalloprotease 9 (MMP‐9) (Player et al., 2014). In addition, mechanical loading of human‐craniofacial‐muscle‐derived myotubes embedded within a sponge of 3D collagen networks induced significant increases in MMP‐2 expression (Auluck, Mudera, Hunt, & Lewis, 2005). However, a more detailed characterization of the molecular and phenotypic hypertrophic effects of mechanical loading in 3D engineered muscle has yet to be fully elucidated.

Thus, the aim of the current investigation was to examine the effect of mechanically loading 3D tissue‐engineered skeletal muscle on mediating skeletal muscle hypertrophy. Specifically, progressive mechanical overload of engineered muscle was used to determine its temporal effects on myotube hypertrophy, tissue function, mTOR signalling, and well‐known hypertrophic and atrophic transcripts (IGF‐1, MMP‐2, MMP‐9, MAFbx, and MuRF‐1). We hypothesised that progressive mechanical overload would induce an increase in specific mechano‐regulated gene transcripts and posttranslational responses similar to those observed in vivo, ultimately inducing myotube hypertrophy and augmenting maximal force production.

## 2 MATERIALS AND METHODS

### 2.1 Cell culture

The immortalized C2C12 murine skeletal muscle myoblast cell line (RRID: CVCL_0188; ECACC, Sigma Aldrich, UK) was used for all experiments herein, and, as such, this study was exempt from receiving full approval from the Loughborough University ethics committee. C2C12 murine myoblast cells were sub‐cultured in T80 flasks (Nunc; Thermo Fisher Scientific, UK) and incubated in a humidified 5% CO_2_ atmosphere. Cells were cultured in growth media (GM: high glucose Dulbecco's modified Eagle's media [DMEM, Thermo Fisher Scientific, UK]), supplemented with 20% (v/v) fetal bovine serum (FBS, PAN Biotech, Germany) and 1% (v/v) penicillin‐streptomycin (P/S; Gibco, UK). GM was changed every 24 hr until 80% confluence was reached, before passage. For all experimentation, cells had undergone fewer than 12 passages.

### 2.2 Fabrication of tissue engineering skeletal muscle

**Figure 1:**
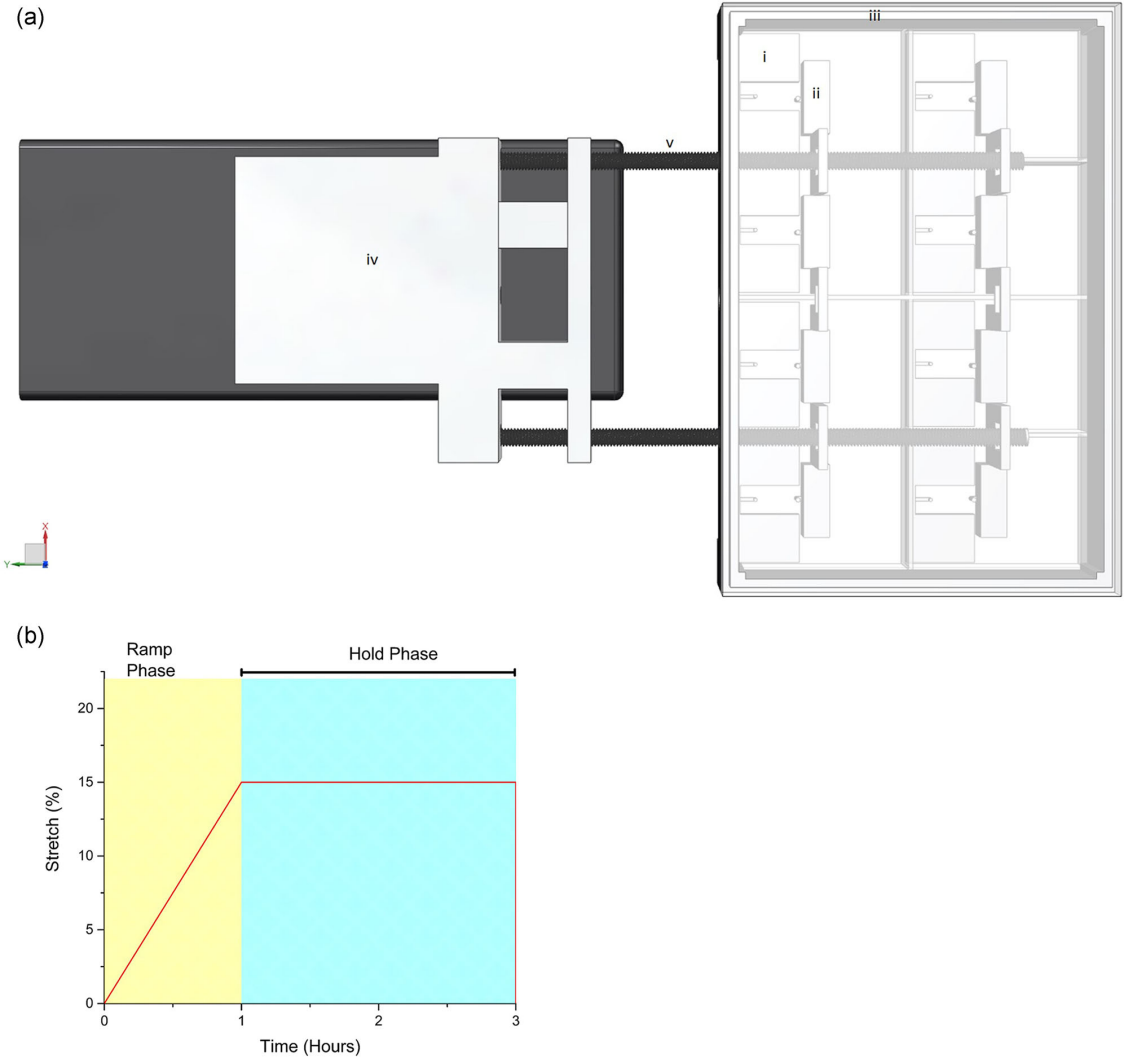
Experimental diagrams of: (a) CAD model of the MSB used for mechanical overload, i) tissue engineered skeletal muscle mould, ii) mechanical loading bar, iii) culture plate border containing three loading portals, iv) movement arm containing two rods attached to loading bar, and v) stepper motor. (b) Graphical illustration of progressive mechanical overload regime [Color figure can be viewed at wileyonlinelibrary.com]

The printing of the tissue engineering inserts was performed by fused deposition modelling (FDM). Inserts were designed with a twin or single post positioned at the end of an open‐ended rectangular chamber, alongside a matching loading bar (Figure 1). All the relevant standard tessellation language (.stl) files for the designs contained within this manuscript are freely available to download at the following domain: https://figshare.com/projects/3D_Printed_Tissue_Engineering_Scaffolds/36494. Chamber sizes were scaled to match the volume of collagen required for each construct (Table 1). All 3D modelling was completed using computer aided design (CAD) Siemens NX software (version 10), with converted.stl files verified using MiniMagics (Materialise, Belgium). Verified.stl files were processed using the Cura Software for Ultimaker 2+ (version 3.2). Printed inserts were adhered to culture well plates using polydimethylsiloxane (PDMS; Sylgard® 184, Dow Corning) and sterilized with 70% industrial methylated spirits (IMS; Thermo Fisher Scientific) and left to evaporate. All samples were then further sterilised via UV irradiation for 24 hr before use. 3D skeletal muscle was engineered as previously described (Player et al., 2014). Briefly, 10% (v/v) 10× minimum essential medium (MEM; Gibco, UK) was added to 85% type I rat‐tail collagen (First Link, UK; in 0.1 M acetic acid, protein concentration 2.035 mg/ml). The solution was neutralised using 5 M and 1 M sodium hydroxide (NaOH) in a drop‐wise fashion until a colour change (yellow to cirrus pink) was observed. Subsequently, 4 × 10^6^ cells/ml C2C12 murine skeletal myoblast cells (ECAAC, UK) were added in a total volume of 0.1 ml DMEM (Gibco, UK). The collagen/cell solution was pipetted into each 3D printed custom well insert (Table 1). The engineered muscles were then placed in a 37°C humidified incubator with 5% CO_2_ for up to 30 min to set. Subsequently, each 500 µl construct was manually detached using a scalpel blade from the base of the mould. Three milliliter of GM was added to each construct. The engineered muscles were then incubated in GM for 4 days, with media replenished daily. After 4 days in culture, GM was removed and replaced with differentiation media (DM: high glucose DMEM [Sigma Aldrich, UK]), supplemented with 2% horse serum (Sigma Aldrich, UK), and 1% P/S (Gibco, UK), which was changed daily for a further 10 days.

**Table 1:** Internal culture dimensions for each collagen gel insert utilised

Collagen gel volume (µl)	Collagen gel length (mm)	Collagen gel width (mm)	Length: width ratio
500	21	6	3.7
50	9.7	2.8	3.7

*Source*: All.stl files for these inserts are freely available to download at the following domain: https://figshare.com/projects/3D_Printed_Tissue_Engineering_Scaffolds/36494 (https://doi.org/.org/10.17028/rd.lboro.6969851.v1, https://doi.org/.org/10.17028/rd.lboro.6969710.v1, https://doi.org/.org/10.17028/rd.lboro.6969707.v1).

### 2.3 Experimental design

After 14 days of culture, engineered muscles were floated in DM and were progressively mechanically loaded using a mechanical stimulation bioreactor (MSB). The MSB is a device programmed to apply progressive mechanical load (Figure 1) to several engineered muscles, using a floatation bar attached to two pins mounted to a stepper motor (Parker). The progressive load regime (Figure 1) described a continuous increasing load to achieve 15% stretch over a 1‐hr period; thereafter engineered muscle was left under tension (15% stretch) for a further 2 hr, enabling maximal mechanical load upon the myotubes. Engineered muscles were collected at differing timepoints respective to outcome measures. Non‐loaded engineered muscle will be referred to as CON.

### 2.4 RNA extraction and quantitative reverse transcription PCR (RT‐qPCR)

After mechanical stimulation (0, 21, and 45 hr after load), engineered muscles were homogenised in 500 µl of TRizol^™^ (Invitrogen; Thermo Fisher Scientific, UK) using TissueLyser beads (Qiagen, UK) and disrupted for 3 × 120 s at 20 Hz using the TissueLyser II (QIAGEN^™^, UK). RNA extraction was performed using the TRIzol method according to the manufacturer's instructions (Sigma Aldrich, UK). RNA concentration and quality were assessed by UV spectroscopy at optical densities of 260 and 280 nm using a Nanodrop 2000 spectrophotometer (Thermo Fisher Scientific). One‐step RT‐PCR amplifications were carried out using the Quantifast SYBR Green RNA‐to‐CT 1 step kit, loading 20 ng of RNA per reaction (Qiagen, Germany) on a ViiA7^™^ Real‐Time PCR System (Applied bio‐system, Life Technologies), and analyzed using ViiA7^™^ RUO software. The RT‐PCR procedure was as follows: 50°C, 10 min (for cDNA synthesis); 95°C, 5 min (transcriptase inactivation); followed by 95°C, 10 s (denaturation); 60°C, 30 s (annealing/extension) for 40 cycles. Melt curve analyses were performed to determine and omit nonspecific amplification or primer‐dimer samples. Relative gene expressions were calculated using the comparative *C*_T_ (ΔΔ*C*_T_) equation (Schmittgen & Livak, 2008) for normalized expression ratios; relative expression was calculated as $2^{{-\Delta \Delta C}_{T}}$, where *C*_T_ is representative of the threshold cycle. POLR2B (RPII β) was used as the housekeeping gene in all RT‐PCR assays, and data were calibrated to a single control (CON) sample from each independent experiment. Primer information can be found in Table 2. All reactions were performed in triplicate.

**Table 2:** Primer sequences for the housekeeping gene POLR2B, IGF‐1, as well as MMP‐2 and MMP‐9 which were used as markers of muscle hypertrophy and the E3 ubiquitin ligases MuRF‐1 and MAFbx which were used as markers of muscle atrophy

	Forward (5′‐3′)	Reverse (5′‐3′)	Product length	NCBI reference sequence
POLR2B	GGTCAGAAGGGAACTTGTG	GCATCATTAAATGGAGTAG	148	NM_153798.2
IGF‐1	GCTTGCTCACCTTTACCAGC	TTGGGCATGTCAGTGTGG	280	NM_010512
MMP‐2	GAGATCTTCTTCTTCAAGGAC	AATAGACCCAGTACTCATTCC	62	NM_008610.2
MMP‐9	CTTCCAGTACCAAGACAAAG	ACCTTGTTCACCTCATTTTG	76	NM_013599.2
MAFbx	CCCAAGGAAAGAGCAGTATGGAGA	GGGTGAAAGTGAAACGGAGCA	134	NM_026346.3
MuRF‐1	AAACAGGAGTGCTCCAGTCGG	CGCCACCAGCATGGAGATACA	67	NM_001039048.2

Abbreviations: IGF‐1, insulin like growth factor‐1; MMP‐2, matrix metalloprotease‐2; MMP‐9, matrix metalloprotease‐9; MuRF‐1, muscle ring finger protein‐1; MAFbx, muscle atrophy F box; POLR2B, RNA polymerase II beta.

### 2.5 Immunoblotting

Due to the known scalability of the engineered muscles (Capel et al., 2019), 500 µl engineered muscles were utilized due to protein quantity constraints within the 50 µl engineered muscles. Engineered muscles were removed 0 hr, 30 min, 3 hr, 6 hr, and 21 hr after mechanical loading, washed three times with ice‐cold phosphate buffered saline (PBS), and then immediately snap‐frozen in liquid nitrogen, before storage at − 80°C. Samples were homogenized as described above, in RIPA lysis buffer (Thermo Fisher Scientific, UK), containing a protease and phosphatase inhibitor, and then allowed to rotate for 60 min at 4°C. The samples were then centrifuged at 12,000×*g* for 10 min. The supernatant was aspirated and placed into a fresh Eppendorf tube. Proteins were quantified using the Pierce^™^ 660 nm protein assay according to the manufacturer's instructions (Thermo Fisher Scientific). Cell lysates were mixed in 4× Laemmli buffer (Bio‐Rad Laboratories), and boiled for 5 min at 95°C, then placed on ice. Ten microgram of each sample was loaded into precast gels (Bio‐Rad) and separated by electrophoresis.

Proteins were transferred on to nitrocellulose membranes and blocked in 5% blocking grade milk (Bio‐Rad) at 4°C for 1 hr. After membrane incubation, the membrane was washed three times with Tris‐buffered saline+0.1% Tween (TBST), and incubated with the appropriate primary antibody. All antibodies were purchased from Cell Signalling Technologies. Specifically, phosphor p70S6 kinase ^Thr389^ (#9234, 1:1,000, RRID: AB_2269803), phosphor 4EBP‐1 ^Thr37/46^ (#2855, 1:2,000, RRID: AB_560835), or phosphor Akt ^Ser 473^ (#4060, 1:2,000, RRID: AB_2315049) was re‐suspended in milk/bovine serum albumin (BSA) and incubated overnight at 4°C. Membranes were washed three times in TBST and incubated with Horseradish peroxidase conjugated antirabbit secondary antibodies (#7074, 1:2,000, RRID: AB_2099233), diluted in TBST and 2% skimmed milk powder for 1 hr at room temperature (RT). The membrane was then washed in TBST three times for 5 min and incubated for 5 min in the dark with enhanced chemiluminescence (ECL) reagent (Thermo Fisher Scientific, UK). Detection of the proteins was visualized with chemiluminescence (chemiDoc^™^MP System; Bio‐Rad Laboratories) with Quantity One 4.6.8 analysis software (Bio‐Rad). Phosphorylation levels were normalized to the total protein loaded, determined via staining of the gels with Coomassie blue (Gel Code; Thermo Fisher Scientific, UK). Data points are presented as a fold change compared to their relevant control samples (CON) in each experimental repeat (*n* = 7).

### 2.6 Histochemistry

After mechanical stimulation, engineered muscles were washed in PBS and fixed via dropwise addition of ice‐cold methanol/acetone solution. The engineered muscles were removed from their pins before being permeabilized with 1× Tris buffered saline (TBS: 0.5 mM) and 0.2% Triton x‐100 (Thermo Fisher Scientific, UK) for 30 min. Engineered muscles were then incubated in Rhodamine Phalloidin (1:500, RRID: AB_2572408; Thermo Fisher Scientific, UK) and 4,6‐diamidino2‐phenylindole (1:2,000, DAPI, RRID: AB_2307445; Thermo Fisher Scientific, UK) for 2 hr at RT in the dark before being washed thoroughly with TBS. Samples were dried and mounted onto polysine adhesion slides (Thermo Fisher Scientific, UK) and sealed with glass cover slides (22 × 50 mm; Menzel, UK) using Fluoromount^™^ aqueous mounting medium (Sigma Aldrich, UK). Images were captured using a Leica DM2500 fluorescent microscope at ×40 magnifications and morphological analysis was carried out using the image processing package FIJI (ImageJ, SciJava, RRID: SCR_003070), with a minimum of five images analyzed per engineered muscle. Within each image, the nuclei within the myotubes and total nuclei were counted. The fusion efficiency of each construct was calculated as the number of nuclei incorporated into myotubes, expressed as a percentage of the total number of nuclei. A myotube was defined as a cell possessing three or more nuclei.

### 2.7 Assessment of functional muscle

To determine if mechanical loading produced an increase in maximal tetanic skeletal muscle force, 21 and 45 hr after mechanical loading, engineered muscles were immersed in 3 ml Krebs‐Ringer‐HEPES buffer (KRH; 10 mM HEPES, 138 mM NaCl, 4.7 mM KCL, 1.25 mM CaCl_2_, 1.25 mM MgSO_4_, 5 mM Glucose, and 0.05% bovine serum albumin in dH_2_0) and attached to a force transducer (403A; Aurora Scientific Ltd., UK). Wire electrodes were positioned either side of the construct to allow for electric field stimulation. Impulses were generated using LabVIEW software (RRID: SCR_014325; National Instruments, Berkshire, UK) connected to a custom‐built amplifier. Maximal twitch force was determined using a single 3.6 V/mm, 1.2 ms impulse, and maximal tetanic force was measured using a 1 s pulse train at 100 Hz and 3.6 V/mm, generated using LabVIEW 2012 software (National Instruments, UK). Where possible, twitch and tetanus data were derived from three contractions per construct. Data were acquired using a Powerlab system (ver. 8/35, RRID: SCR_001620) and associated software (Labchart 8; AD Instruments, UK).

### 2.8 Statistical analysis

All statistical analysis was performed using SPSS software version 23 (RRID: SCR_002865; SPS Inc., Chicago, IL). Normality of distribution and homogeneity of variance in all data sets were determined using a Shapiro‐Wilk test and Levine's test, respectively. Data sets were then appropriately analyzed using a one‐way ANOVA with an Fishers Least Significant Difference (LSD) post hoc test. A Kruskal–Willis test was performed where data were not normally distributed. All data are presented as mean ± standard deviation (SD).

## 3 RESULTS

### 3.1 Regulation of hypertrophic and atrophic related genes following mechanical overload

**Figure 2:**
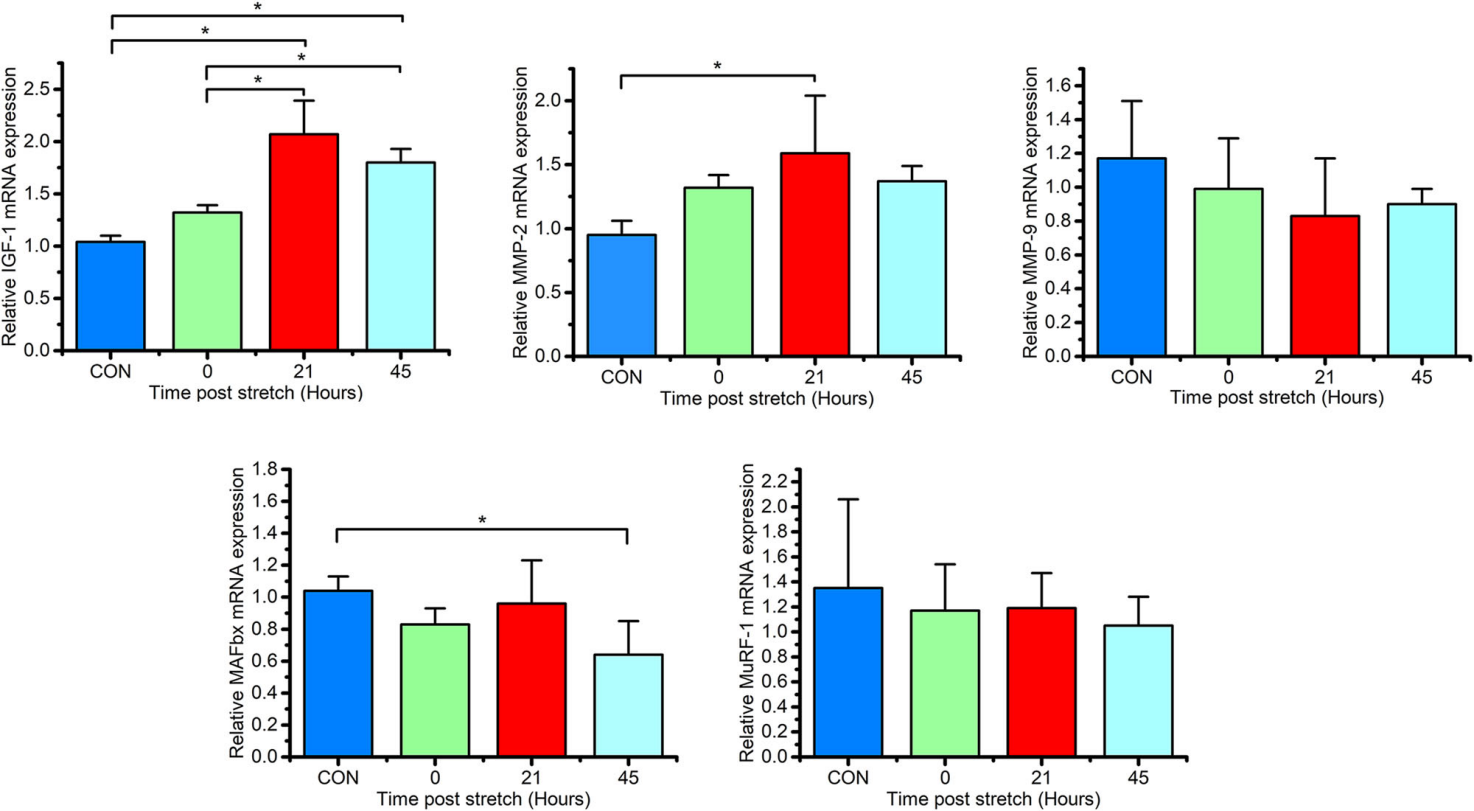
Relative mRNA expression of IGF‐1, MMP‐2, MMP‐9, MAFbx, and MuRF‐1 after mechanical overload (Control [CON] [n = 4], 0 [*n* = 3] ,21 [*n* = 6] and 45 hr [*n* = 4]). Values are normalized to POLR2B and made relative to static engineered muscles (CON). Data are expressed as mean ± *SD*. Significant values are identified using * where a significance of *p* ≤ 0.05 was achieved. IGF‐1, insulin like growth factor‐1; MMP‐2, matrix metalloprotease‐2; MMP‐9, matrix metalloprotease‐9; MuRF‐1, muscle ring finger protein‐1; MAFbx, muscle atrophy F box; mRNA, messenger RNA; POLR2B, RNA polymerase II beta; SD, standard deviation [Color figure can be viewed at wileyonlinelibrary.com]

3D engineered skeletal muscle was mechanically loaded for 3 hr as described in Figure 1, and the mRNA levels of candidate hypertrophy‐related genes were examined over the subsequent 48 hr (Figure 2). Timepoints of 21 and 45 hr after stretch were examined to determine gene expression changes 24 and 48 hr after the onset of stretch. Expression of IGF‐1 mRNA within mechanically loaded engineered muscle was unchanged immediately after loading (1.32 ± 0.07), but significantly upregulated 21 and 45 hr after loading compared to nonloaded counterparts (CON; 2.07 ± 0.32, 1.80 ± 0.13, and 1.04 ± 0.09, respectively; *p *< .001). We observed temporal increases in MMP‐2, with mRNA expression statistically augmented at 21 hr after loading compared to CON (*p *< .01). Alternatively, MMP‐9 mRNA expression was left unaltered after mechanical overload, although a nonsignificant downward trend was apparent (*p *= .359). MuRF‐1 and MAFbx mRNA levels were also investigated after mechanical loading as markers of muscle breakdown. A nonsignificant downward trend was observed in MuRF‐1 mRNA expression after mechanical loading compared to CON (*p *= .798), whilst, in contrast, a decline in MAFbx mRNA expression was observed after all timepoints post mechanical ramp loading, reaching statistical significance 45 hr after mechanical loading, with a decline of ~40% (*p *< .05)—suggesting a degree of suppression of the ubiquitin proteasome system after mechanical loading.

### 3.2 Mechanical loading increases the phosphorylation of Akt and mTORC1 targets p70S6K and 4EBP‐1

**Figure 3:**
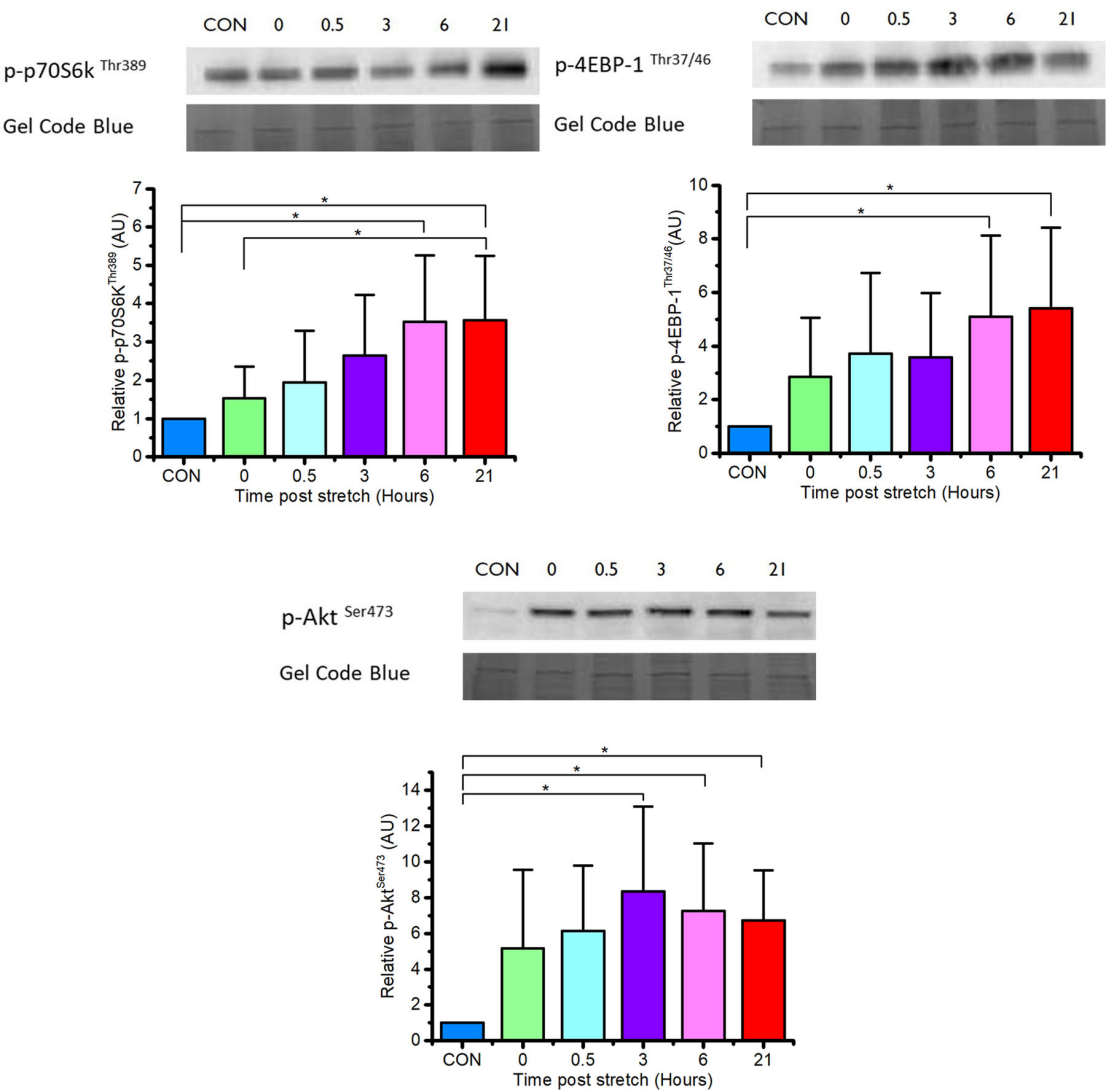
Phosphorylation of p70S6 kinase, 4EBP‐1, and Akt at various time points after mechanical loading. Phosphorylated p70S6 kinase, 4EBP‐1, and Akt is normalized to the Gel Code blue stain reagent stain. Data are expressed as mean ± *SD* for *n* = 7 engineered muscles. Significant values are identified using * where a significance of *p* ≤ .05 was achieved. SD, standard deviation [Color figure can be viewed at wileyonlinelibrary.com]

Due to its importance in cellular growth, members of the mTORC1 pathway Akt, p70S6K, and 4EBP‐1 were investigated after mechanical loading (Figure 3). Phosphorylation of Akt was significantly upregulated 3, 6, and 21 hr after loading when compared to CON (*p *< .05), with peak phosphorylation at 3 hr. Thereafter, Akt remained elevated 6 and 21 hr after loading. Peak phosphorylation of both p70S6K and 4EBP‐1 was evident at 6 hr after mechanical loading and remained elevated at 21 hr relative to CON (*p *< .05).

### 3.3 Increased mechanical loading is associated with myotube hypertrophy in tissue engineered skeletal muscle

**Figure 4:**
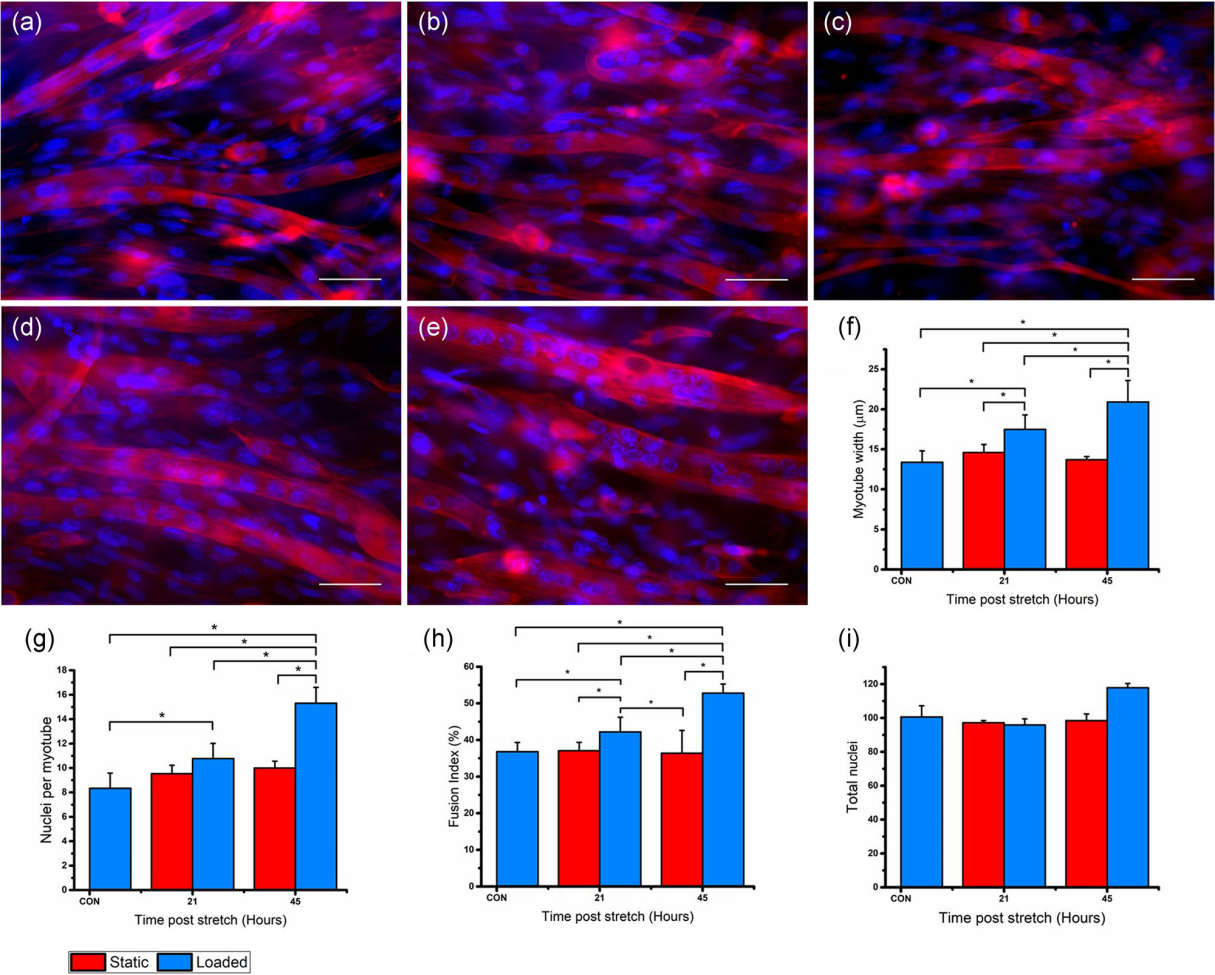
Fluorescent staining of the nucleic DNA (blue) and the actin cytoskeleton (red) in engineered muscles with (×40 magnification) CON, 21 and 45 hr postmechanical load (a) myotubes subject to no stretch (CON; *n* = 6), (b) myotubes subject to no stretch 21 hr post (*n* = 3), (c) myotubes subject to no stretch 45 hr post (*n* = 3), (d) myotubes 21 hr after load (*n* = 6), (e) myotube 45 hr after load (*n* = 6), (f) Myotube width (μm), (g) nuclei per myotube, (h) fusion index (%), and (i) total nuclei of CON(no stretch), 21 and 45 hr after mechanical loading (blue bars represent static engineered muscles and red bars represent loaded engineered muscles). Scale bar represents 50 μm. Significantly different values are identified using*. Data representative of three experimental repeats and presented as mean ± *SD*. SD, standard deviation [Color figure can be viewed at wileyonlinelibrary.com]

To discern whether the apparent augmentation of anabolic gene expression and signalling are translated into muscle growth after mechanical loading of engineered muscle, we next investigated myotube size and related morphology (Figure 4). Average myotube widths within engineered muscles were significantly increased when measured 21 and 45 hr after the cessation of mechanical load, compared to CON at 14 days (*p *< .001). A significant increase in nuclei per myotube was observed both 21 and 45 hr after mechanical loading compared to CON at day 14 (*p *< .01), suggesting enhanced fusion of myoblasts into existing myotubes. In support of this, enhanced fusion index was reported 21 and 45 hr after mechanical loading compared to controls at day (*p *< .01). No significant differences in myotube width, fusion efficiency, nuclei per myotube, and total nuclei were apparent after an additional period of static differentiation, where no loading was administered, compared to CON (21 and 45 hr; *p* > .05). Together, these data suggest that mechanical loading induced a regenerative and hypertrophic response in 3D engineered skeletal muscle.

### 3.4 Mechanical loading enhances maximal contractile force production

**Figure 5:**
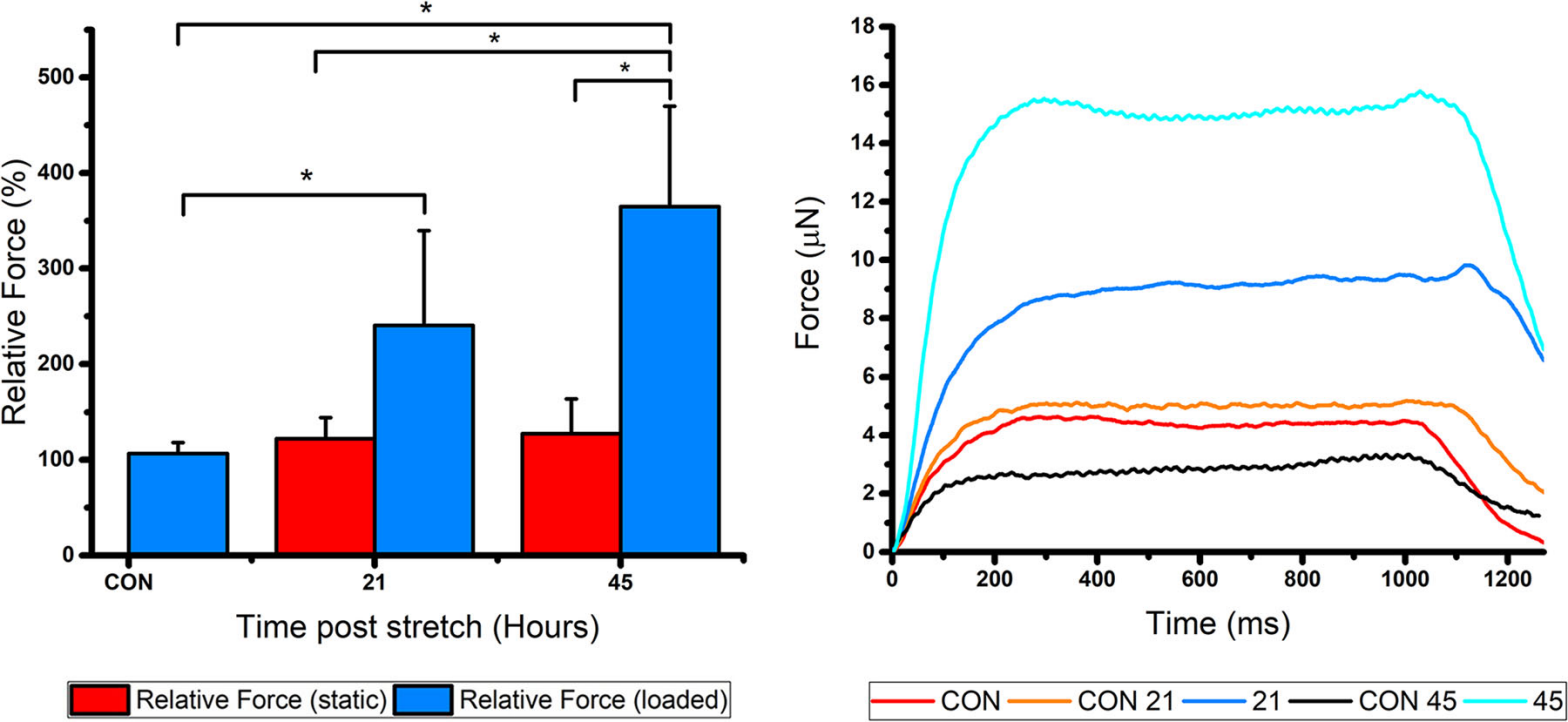
Maximal contractile force from 50 µl engineered skeletal muscle cultures at each time point 21 and 45 hr after mechanical loading. All cultures were compared to CON at day 14 within individual experimental repeats to calculate relative force. Data are representative of three experimental repeats and expressed as mean ± *SD*, for *n* = 9 engineered muscles. SD, standard deviation [Color figure can be viewed at wileyonlinelibrary.com]

Maximal isometric force was measured in 3D engineered skeletal muscle after mechanical loading (Figure 5). Compared to non‐loaded muscles, relative force production increased in a step‐wise fashion 21 (140%) and 45 hr (265%) after the cessation of stimulation compared to CON (*p *< .01). By contrast, there was no augmentation of muscle function as a result of the additional time in culture in the absence of mechanical loading (*p *= 1.00).

## 4 DISCUSSION

Mechanical loading in both humans and animals result in skeletal muscle hypertrophy and increased maximal force production, with both components mediated through alterations in cellular signalling and gene expression. To date, research is lacking an appropriate experimental in vitro model to study mechanical loading. In the present study, 3D tissue engineered skeletal muscle was mechanically loaded using a bespoke bioreactor. Mechanical loading temporally activated mTORC1 signalling, gene expression transcripts associated with skeletal muscle hypertrophy, alongside myotube hypertrophy and increases in maximal force production in the days following mechanical loading.

Previously, 3D engineered skeletal muscle has been mechanically loaded during myoblast differentiation (Boonen et al., 2010; Moon du et al., 2008; Okano & Matsuda, 1997), establishing improvements in myotube development and morphology. Our laboratory has previously mechanically loaded highly differentiated 3D engineered muscle and observed a gene expression profile associated with muscle growth and regeneration; however phenotypic effects were not investigated (Player et al., 2014). In the present study, we measured the mRNA levels of candidate genes based on literature and our previous observations, demonstrating significant increases in IGF‐1 mRNA expression 21 hr after the cessation of loading. Although increases in IGF‐1 mRNA were apparent after loading, the magnitude was somewhat lower than that we have observed in our previous experiments (Player et al., 2014), but similar to the effects of muscle loading in humans (Bamman et al., 2001; Deldicque et al., 2005).

We also examined the mRNA levels of matrix metalloprotease 2 and 9 (MMP‐2, 9), both of which are pivotal to skeletal muscle development, repair, and adaptive modifications induced by physical exercise (Carmeli, Moas, Lennon, & Powers, 2005; Rullman et al., 2007). In vivo mechanical loading including those mimicking eccentric (Heinemeier et al., 2007), high impact (Carmeli et al., 2005; Rullman et al., 2007) and endurance exercise (Rullman et al., 2009), have produced large increases in the local production of MMP‐2 and MMP‐9 in skeletal muscle. In the present study, it was observed that MMP‐2 mRNA expression was significantly upregulated 21 hr after loading. However, MMP‐9 mRNA was left unaltered, in contrast to our previously published work, in which large increases in MMP‐9 mRNA expression were observed (Player et al., 2014). Discrepancies in the gene expression profiles were highlighted maybe due to differences in engineered skeletal muscle size, design (i.e. flotation bar vs fixed pins), differentiation status, and experimental protocol. Nevertheless, the stimulated increase in MMP‐2 mRNA expression after loading is indicative of enhanced matrix remodelling, enabling subsequent myotube formation and hypertrophy (Choi et al., 2002).

MuRF‐1 and MAFbx are E3 ubiquitin ligases are understood to act through the ubiquitin proteasome pathway (Glass, 2003; Yang, Jemiolo, Trappe, & Proteolytic, 2006), leading to muscle protein breakdown. In vivo, following exercise, MAFbx and MuRF‐1 have been shown to react in a bi‐phasic manner. Describing an initial increase in MuRF‐1 mRNA expression, followed by a decrease in MAFbx mRNA expression at 8 and 12 hr after resistance exercise, with all proteolytic mRNA expressions returning to pre‐exercise values 24 hr after exercise (Louis, Raue, Yang, Jemiolo, & Trappe, 2007). We were able to determine temporal changes in MuRF‐1 and MAFbx mRNAs, identifying a significant decrease in ubiquitin ligase MAFbx 45 hr after mechanical loading, with no significant changes in MuRF‐1 mRNA expression. Overall, the time‐dependent gene expression profile from mechanically loaded engineered skeletal muscle is indicative of a molecular hypertrophic response.

Muscle hypertrophy is mechanistically underpinned by mTORC1 through activation of downstream targets p70S6 kinase (p70S6K) and 4EBP‐1 (eIF‐4E‐ binding protein), triggering an increase in translation initiation, elongation, and ribosome biogenesis (Kubica, Bolster, Farrell, Kimball, & Jefferson, 2005; Marabita et al., 2016). Both human and animal studies have successfully determined that the phosphorylation of p70S6K and 4EBP‐1 is rapidly and transiently upregulated within 1 hr after resistance exercise (McGlory et al., 2014; Ogasawara et al., 2016), often reducing to baseline within 24 hr (Ogasawara et al., 2016). We show here that mechanically loaded engineered muscle exhibits step‐wise increases in the phosphorylation of both downstream mTOR targets, with peak phosphorylation 21 hr after the loading period. Whilst we cannot account for differences in the temporal activation of mTORC1 signalling in our system compared to in vivo data, a small but significant increase in the hyperphosphorylation status of p70S6k and 4EBP‐1 16 hr after resistance exercise has previously been reported in rats (Kubica et al., 2005). To further examine the nature of mTORC1 activation, we also assessed Akt phosphorylation after mechanical loading. Indeed, our observation that Akt phosphorylation was stimulated by loading suggests that the activation of mTORC1 was, at least in part, due to a growth‐factor‐mediated pathway. This is in contrast to specific data in humans where Akt phosphorylation has been shown to decrease after resistance exercise (Deldicque et al., 2008), and deletion of the IGF‐1 receptor or the pharmacological inhibition of PI3 Kinase (which are upstream positive regulators of Akt) does not impair loading‐induced mTORC1 activation and muscle growth (Hornberger et al., 2004; Spangenburg, Le Roith, Ward, & Bodine, 2008). Nevertheless, in humans, Akt phosphorylation has also been observed to elevate as early as 60 min after resistance exercise (Dreyer et al., 2006), comparable to the current investigation and remains elevated up to 24 hr after resistance exercise (Mayhew, Kim, Cross, Ferrando, & Bamman, 2009). As such, in our system, both loading and growth factors (presumably IGF‐1) likely contribute to the activation of mTORC1.

A noteworthy finding from the present study was that after mechanical loading, myotube fusion, and size both increased, a characteristic of myotube hypertrophy. Mechanical stretch of in vitro bioengineered muscle has previously been shown to improve both the orientation and density of rodent myofibers cultured in collagen gels (Okano & Matsuda, 1997). Yet, mechanical loading of post‐mitotic engineered muscle and its effects on myotube development are yet to be fully elucidated. We show here that myotube hypertrophy in the days after mechanical loading is associated with increased myonuclear number and increased fusion index. As such, this myonuclear addition resembles the satellite‐cell‐mediated myogenic response observed in native tissue after exercise (Bruusgaard, Johansen, Egner, Rana, & Gundersen, 2010), and likely contributes, alongside IGF‐1‐Akt‐mTORC1 activation, to the enhancement in myotube size. Finally, we were able to establish enhanced functionality of the mechanically loaded engineered muscle through an increase in maximal contractile force production compared to unloaded controls. Recently, the use of electrical stimulation to enable a more mature adult phenotype and increase functionality in engineered muscle has been established (Khodabukus & Baar, 2012; Khodabukus et al., 2019); however research on the use of mechanical loading and engineered muscle functionality and hypertrophy is limited. Although the present model produced force lower than that previously published (Martin et al., 2017; Shimizu, Genma, Gotou, Nagasaka, & Honda, 2017), mechanical overload of the postmitotic engineered skeletal muscle led to significant increases in maximal tetanic force both 21 and 45 hr post, signifying increased functionality of the tissue‐engineered skeletal muscles. Increases in contractile force in systems such as this can be attributed to several factors such as myotube hypertrophy, maturation, and rearrangements within the actin cytoskeleton (Khodabukus & Baar, 2012; Powell et al., 2002). Whilst we cannot discount multiple factors contributing to enhanced contractile force after mechanical loading, we were unable to depict obvious differences in the cytoskeleton staining pattern between stimulated and control engineered muscles, while myotube size was significantly augmented.

In conclusion, we have described the molecular response and morphological and functional adaptation to mechanical loading in mature tissue engineered skeletal muscle. Altogether, the observed increase in IGF‐1 mRNA, phosphorylation of Akt, p70S6K, and 4EBP‐1, alongside myotube hypertrophy and an increase in functionality of the engineered muscle clearly display an in vitro model of skeletal muscle hypertrophy, similar to that seen in vivo after mechanical loading. As such, this system is amenable to future investigations concerned with enhancing our understanding of skeletal muscle plasticity in health and disease.

## FUNDING INFORMATION

The authors would like to acknowledge Loughborough University, the EPSRC EP/L02067X/1, and MRC Centre for Doctoral Training in Regenerative Medicine for funding and support for this study.

## CONFLICT OF INTERESTS

The authors declare that there are no conflict of interests.

## AUTHOR CONTRIBUTIONS

KA performed all experimental work and data analysis and drafted the manuscript. AC significantly contributed to all the CAD designs used throughout the manuscript. AC, DP, and NM contributed significantly to experimental design and critically reviewed the manuscript. ML conceived the concept of work, contributed to experimental design, and reviewed the manuscript.

## References

[jcp28923-bib-0001] Adams, G. R. , Hather, B. M. , Baldwin, K. M. , & Dudley, G. A. (1993). Skeletal muscle myosin heavy chain composition and resistance training. Journal of Applied Physiology, 74(2), 911–915. http://hdl.handle.net/2060/19970022797 8458814 10.1152/jappl.1993.74.2.911

[jcp28923-bib-0002] Auluck, A. , Mudera, V. , Hunt, N. P. , & Lewis, M. P. (2005). A three‐dimensional in vitro model system to study the adaptation of craniofacial skeletal muscle following mechanostimulation. European Journal of Oral Sciences, 113(3), 218–224. 10.1111/j.1600-0722.2005.00215.x 15953246

[jcp28923-bib-0003] Bamman, M. M. , Shipp, J. R. , Jiang, J. , Gower, B. A. , Hunter, G. R. , Goodman, A. , & Urban, R. J. (2001). Mechanical load increases muscle IGF‐I and androgen receptor mRNA concentrations in humans. American Journal of Physiology. Endocrinology and Metabolism, 280(3), E383–E390. https://doi.org/papers3://publication/uuid/1FE63A32‐B42C‐4A25‐98C6‐CF796BFCF14B 11171591 10.1152/ajpendo.2001.280.3.E383

[jcp28923-bib-0004] Bodine, S. C. (2006). mTOR signaling and the molecular adaptation to resistance exercise. Medicine and Science in Sports and Exercise, 38(11), 1950–1957. 10.1249/01.mss.0000233797.24035.35 17095929

[jcp28923-bib-0005] Bodine, S. C. (2013). Disuse‐induced muscle wasting. International Journal of Biochemistry and Cell Biology, 45(10), 2200–2208. 10.1016/j.biocel.2013.06.011 23800384 PMC3856924

[jcp28923-bib-0006] Bodine, S. C. , Stitt, T. N. , Gonzalez, M. , Kline, W. O. , Stover, G. L. , Bauerlein, R. , & Yancopoulos, G. D. (2001). Akt/mTOR pathway is a crucial regulator of skeletal muscle hypertrophy and can prevent muscle atrophy in vivo. Nature Cell Biology, 3(11), 1014–1019. 10.1038/ncb1101-1014 11715023

[jcp28923-bib-0007] Boonen, K. J. M. , Langelaan, M. L. P. , Polak, R. B. , van der Schaft, D. W. J. , Baaijens, F. P. T. , & Post, M. J. (2010). Effects of a combined mechanical stimulation protocol: Value for skeletal muscle tissue engineering. Journal of Biomechanics, 43(8), 1514–1521. 10.1016/j.jbiomech.2010.01.039 20189177

[jcp28923-bib-0008] Bruusgaard, J. C. , Johansen, I. B. , Egner, I. M. , Rana, Z. A. , & Gundersen, K. (2010). Myonuclei acquired by overload exercise precede hypertrophy and are not lost on detraining. Proceedings of the National Academy of Sciences, 107(34), 15111–15116. 10.1073/pnas.0913935107 PMC2930527 20713720

[jcp28923-bib-0009] Capel, A. J. , Rimington, R. P. , Fleming, J. W. , Player, D. J. , Baker, L. A. , Turner, M. C. , & Lewis, M. P. (2019). Scalable 3D printed moulds for human tissue engineered skeletal muscle. Frontiers in Bioengineering and Biotechnology, 7, 20 10.3389/FBIOE.2019.00020 30838203 PMC6383409

[jcp28923-bib-0010] Carmeli, E. , Coleman, R. , & Reznick, A. Z. (2002). The biochemistry of aging muscle. Experimental Gerontology, 37(4), 477–489. 10.1016/S0531-5565(01)00220-0 11830351

[jcp28923-bib-0011] Carmeli, E. , Moas, M. , Lennon, S. , & Powers, S. K. (2005). High intensity exercise increases expression of matrix metalloproteinases in fast skeletal muscle fibres. Experimental Physiology, 90, 613–619. 10.1113/expphysiol.2004.029462 15833756

[jcp28923-bib-0012] Cheema, U. , Brown, R. , Mudera, V. , Shi, Y. Y. , Mcgrouther, G. , & Goldspink, G. (2005). Mechanical signals and IGF‐I gene splicing in vitro in relation to development of skeletal muscle. Journal of Cellular Physiology, 202(1), 67–75. 10.1002/jcp.20107 15389530

[jcp28923-bib-0013] Cheema, U. , Yang, S. , Mudera, V. , Goldspink, G. G. , & Brown, R. A. (2003). 3‐D in vitro model of early skeletal muscle. Development, 54(3), 226–236. 10.1002/cm.10095 12589681

[jcp28923-bib-0014] Choi, H. R. , Kondo, S. , Hirose, K. , Ishiguro, N. , Hasegawa, Y. , & Iwata, H. (2002). Expression and enzymatic activity of MMP‐2 during healing process of the acute supraspinatus tendon tear in rabbits. Journal of Orthopaedic Research, 20(5), 927–933. 10.1016/S0736-0266(02)00016-5 12382955

[jcp28923-bib-0015] Deldicque, L. , Atherton, P. , Patel, R. , Theisen, D. , Nielens, H. , Rennie, M. , & Francaux, M. (2008). Effects of resistance exercise with and without creatine supplementation on gene expression and cell signaling in human skeletal muscle. Journal of Applied Physiology, 104, 371–378. 10.1152/japplphysiol.00873.2007 18048590

[jcp28923-bib-0016] Deldicque, L. , Louis, M. , Theisen, D. , Nielens, H. , Dehoux, M. , Thissen, J. P. , & Francaux, M. (2005). Increased IGF mRNA in human skeletal muscle after creatine supplementation. Medicine and Science in Sports and Exercise, 37(5), 731–736. 10.1249/01.MSS.0000162690.39830.27 15870625

[jcp28923-bib-0017] Dennis, R. G. , & Kosnik, P. E., II (2000). Excitability and isometric contractile properties of mammalian skeletal muscle constructs engineered in vitro. In Vitro Cellular & Developmental Biology ‐ Animal, 36(5), 327–335. 10.1290/1071-2690(2000)036<0327:EAICPO>2.0.CO;2 10937836

[jcp28923-bib-0018] Dons, B. , Bollerup, K. , Bonde‐Petersen, F. , & Hancke, S. (1979). The effect of weight‐lifting exercise related to muscle fiber composition and muscle cross‐sectional area in humans. European Journal of Applied Physiology and Occupational Physiology, 40(2), 95–106. 10.1007/BF00421155 428373

[jcp28923-bib-0019] Dreyer, H. C. , Fujita, S. , Cadenas, J. G. , Chinkes, D. L. , Volpi, E. , & Rasmussen, B. B. (2006). Resistance exercise increases AMPK activity and reduces 4E‐BP1 phosphorylation and protein synthesis in human skeletal muscle. Journal of Physiology, 576(2), 613–624. 10.1113/jphysiol.2006.113175 16873412 PMC1890364

[jcp28923-bib-0020] Glass, D. J. (2003). Signalling pathways that mediate skeletal muscle hypertrophy and atrophy. Nature Cell Biology, 5(2), 87–90. 10.1038/ncb0203-87 12563267

[jcp28923-bib-0021] Goodman, C. A. , Frey, J. W. , Mabrey, D. M. , Jacobs, B. L. , Lincoln, H. C. , You, J. ‐S. , & Hornberger, T. A. (2011). The role of skeletal muscle mTOR in the regulation of mechanical load‐induced growth. The Journal of Physiology, 589(22), 5485–5501. 10.1113/jphysiol.2011.218255 21946849 PMC3240886

[jcp28923-bib-0022] Heinemeier, K. M. , Olesen, J. L. , Haddad, F. , Langberg, H. , Kjaer, M. , Baldwin, K. M. , & Schjerling, P. (2007). Expression of collagen and related growth factors in rat tendon and skeletal muscle in response to specific contraction types. Journal of Physiology, 582(3), 1303–1316. 10.1113/jphysiol.2007.127639 17540706 PMC2075262

[jcp28923-bib-0023] Hornberger, T. A. , Stuppard, R. , Conley, K. E. , Fedele, M. J. , Fiorotto, M. L. , Chin, E. R. , & Esser, K. A. (2004). Mechanical stimuli regulate rapamycin‐sensitive signalling by a phosphoinositide 3‐kinase‐, protein kinase B‐ and growth factor‐independent mechanism. Biochemical Journal, 380(Pt 3), 795–804. 10.1042/BJ20040274 15030312 PMC1224227

[jcp28923-bib-0024] Huang, Y. ‐C. Y. , Dennis, R. R. G. R. R. G. , Larkin, L. , & Baar, K. (2005). Rapid formation of functional muscle in vitro using fibrin gels. Journal of Applied Physiology, 98(2), 706–713. 10.1152/japplphysiol.00273.2004 15475606

[jcp28923-bib-0025] Kasper, A. M. , Turner, D. C. , Martin, N. R. W. , & Sharples, A. P. (2018). Mimicking exercise in three‐dimensional bioengineered skeletal muscle to investigate cellular and molecular mechanisms of physiological adaptation. Journal of Cellular Physiology, 233(3), 1985–1998. 10.1002/jcp.25840 28158895

[jcp28923-bib-0026] Khodabukus, A. , & Baar, K. (2012). Defined electrical stimulation emphasizing excitability for the development and testing of engineered skeletal muscle. Tissue Engineering Part C: Methods, 18(5), 349–357. 10.1089/ten.tec.2011.0364 22092374

[jcp28923-bib-0027] Khodabukus, A. , Madden, L. , Prabhu, N. K. , Koves, T. R. , Jackman, C. P. , Muoio, D. M. , & Bursac, N. (2019). Electrical stimulation increases hypertrophy and metabolic flux in tissue‐engineered human skeletal muscle. Biomaterials, 198, 259–269. 10.1016/j.biomaterials.2018.08.058. 30180985 PMC6395553

[jcp28923-bib-0028] Khodabukus, A. , Paxton, J. Z. , Donnelly, K. , & Baar, K. (2007). Engineered muscle: A tool for studying muscle physiology and function. Exercise and Sport Sciences Reviews, 35(4), 186–191. 10.1097/jes.0b013e318156df01 17921787

[jcp28923-bib-0029] Kortebein, P. , Symons, T. B. , Ferrando, A. , Paddon‐Jones, D. , Ronsen, O. , Protas, E. , & Evans, W. J. (2008). Functional Impact of 10 days of bed rest in healthy older adults. The Journals of Gerontology Series A: Biological Sciences and Medical Sciences, 63(10), 1076–1081. 10.1093/gerona/63.10.1076 18948558

[jcp28923-bib-0030] Kovanen, V. (2002). Intramuscular extracellular matrix: Complex environment of muscle cells. Exercise and Sport Sciences Reviews, 30(1), 20–25. http://www.ncbi.nlm.nih.gov/pubmed/11800495 11800495 10.1097/00003677-200201000-00005

[jcp28923-bib-0031] Kubica, N. , Bolster, D. E. , Farrell, P. A. , Kimball, S. E. , & Jefferson, L. S. (2005). Resistance exercise increases muscle protein synthesis and translation of eukaryotic initiation factor 2Bϵ mRNA in a mammalian target of rapamycin‐dependent manner. Journal of Biological Chemistry, 280(9), 7570–7580. 10.1074/jbc.M413732200 15591312

[jcp28923-bib-0032] Liu, Y. , Schlumberger, A. , Wirth, K. , Schmidtbleicher, D. , & Steinacker, J. M. (2003). Different effects on human skeletal myosin heavy chain isoform expression: Strength vs. combination training. Journal of Applied Physiology, 94(6), 2282–2288. 10.1152/japplphysiol.00830.2002 12736190

[jcp28923-bib-0033] Lo Presti, R. , Hopps, E. , & Caimi, G. (2017). Gelatinases and physical exercise: A systematic review of evidence from human studies. Medicine, 96(37), e8072 10.1097/MD.0000000000008072 28906407 PMC5604676

[jcp28923-bib-0034] Louis, E. , Raue, U. , Yang, Y. , Jemiolo, B. , & Trappe, S. (2007). Time course of proteolytic, cytokine, and myostatin gene expression after acute exercise in human skeletal muscle. Journal of Applied Physiology, 103, 1744–1751. 10.1152/japplphysiol.00679.2007 17823296

[jcp28923-bib-0035] MacDougall, J. D. , Sale, D. G. , Elder, G. C. , & Sutton, J. R. (1982). Muscle ultrastructural characteristics of elite powerlifters and bodybuilders. European Journal of Applied Physiology and Occupational Physiology, 48(1), 117–126. 10.1007/BF00421171 7199447

[jcp28923-bib-0036] Marabita, M. , Baraldo, M. , Solagna, F. , Ceelen, J. J. M. , Sartori, R. , Nolte, H. , & Blaauw, B. (2016). S6K1 is required for increasing skeletal muscle force during hypertrophy. Cell Reports, 17(2), 501–513. 10.1016/j.celrep.2016.09.020 27705797

[jcp28923-bib-0037] Martin, N. R. W. , Aguilar‐Agon, K. , Robinson, G. P. , Player, D. J. , Turner, M. C. , Myers, S. D. , & Lewis, M. P. (2017). Hypoxia impairs muscle function and reduces myotube size in tissue engineered skeletal muscle. Journal of Cellular Biochemistry, 118(9), 2599–2605. 10.1002/jcb.25982 28294416 PMC5518201

[jcp28923-bib-0038] Martin, N. R. W. , Passey, S. L. , Player, D. J. , Khodabukus, A. , Ferguson, R. A. , Sharples, A. P. , & Lewis, M. P. (2013). Factors affecting the structure and maturation of human tissue engineered skeletal muscle. Biomaterials, 34(23), 5759–5765. 10.1016/j.biomaterials.2013.04.002 23643182

[jcp28923-bib-0039] Mayhew, D. L. , Kim, J. , Cross, J. M. , Ferrando, A. A. , & Bamman, M. M. (2009). Translational signaling responses preceding resistance training‐mediated myofiber hypertrophy in young and old humans. Journal of Applied Physiology, 107(5), 1655–1662. 10.1152/japplphysiol.91234.2008 19589955 PMC2777794

[jcp28923-bib-0040] McGlory, C. , White, A. , Treins, C. , Drust, B. , Close, G. L. , MacLaren, D. P. M. , & Hamilton, D. L. (2014). Application of the [ ‐32P] ATP kinase assay to study anabolic signaling in human skeletal muscle. Journal of Applied Physiology, 116(5), 504–513. 10.1152/japplphysiol.01072.2013 24436296 PMC4116398

[jcp28923-bib-0041] Mikesky, A. E. , Giddings, C. J. , Matthews, W. , & Gonyea, W. J. (1991). Changes in muscle fiber size and composition in response to heavy‐resistance exercise. Medicine and Science in Sports and Exercise, 23(9), 1042–1049. http://www.ncbi.nlm.nih.gov/pubmed/1943624 1943624

[jcp28923-bib-0042] Moon du, G. , Christ, G. , Stitzel, J. D. , Atala, A. , & Yoo, J. J. (2008). Cyclic mechanical preconditioning improves engineered muscle contraction. Tissue engineering. Part A, 14(4), 473–482. 10.1089/tea.2007.0104 18399787

[jcp28923-bib-0043] Mudera, V. , Smith, A. S. T. , Brady, M. A. , & Lewis, M. P. (2010). The effect of cell density on the maturation and contractile ability of muscle derived cells in a 3D tissue‐engineered skeletal muscle model and determination of the cellular and mechanical stimuli required for the synthesis of a postural phenotype. Journal of Cellular Physiology, 225(3), 646–653. 10.1002/jcp.22271 20533296

[jcp28923-bib-0044] Ogasawara, R. , Fujita, S. , Hornberger, T. A. , Kitaoka, Y. , Makanae, Y. , Nakazato, K. , & Naokata, I. (2016). The role of mTOR signalling in the regulation of skeletal muscle mass in a rodent model of resistance exercise. Scientific Reports, 6, 1–12. 10.1038/srep31142 27502839 PMC4977552

[jcp28923-bib-0045] Okano, T. , & Matsuda, T. (1997). Hybrid muscular tissues: Preparation of skeletal muscle cell‐incorporated collagen gels. Cell Transplantation, 6(2), 109–118. http://www.ncbi.nlm.nih.gov/pubmed/9142442 9142442 10.1177/096368979700600204

[jcp28923-bib-0046] Player, D. J. , Martin, N. R. W. , Passey, S. L. , Sharples, A. P. , Mudera, V. , & Lewis, M. P. (2014). Acute mechanical overload increases IGF‐I and MMP‐9 mRNA in 3D tissue‐engineered skeletal muscle. Biotechnology Letters, 36(5), 1113–1124. 10.1007/s10529-014-1464-y 24563297

[jcp28923-bib-0047] Powell, C. A. , Smiley, B. L. , Mills, J. , & Vandenburgh, H. H. (2002). Mechanical stimulation improves tissue‐engineered human skeletal muscle. AJP: Cell Physiology, 283(5), C1557–C1565. 10.1152/ajpcell.00595.2001 12372817

[jcp28923-bib-0048] Reynolds, T. H., IV , Bodine, S. C. , & Lawrence, J. C. (2002). Control of Ser2448 phosphorylation in the mammalian target of rapamycin by insulin and skeletal muscle load. Journal of Biological Chemistry, 277(20), 17657–17662. 10.1074/jbc.M201142200 11884412

[jcp28923-bib-0049] Rommel, C. , Bodine, S. C. , Clarke, B. a , Rossman, R. , Nunez, L. , Stitt, T. N. , & Glass, D. J. (2001). Mediation of IGF‐1‐induced skeletal myotube hypertrophy by PI(3)K/Akt/mTOR and PI(3)K/Akt/GSK3 pathways. Nature Cell Biology, 3(11), 1009–1013. 10.1038/ncb1101-1009 11715022

[jcp28923-bib-0050] Rullman, E. , Norrbom, J. , Strömberg, A. , Wågsäter, D. , Rundqvist, H. , Haas, T. , & Gustafsson, T. (2009). Endurance exercise activates matrix metalloproteinases in human skeletal muscle. Journal of Applied, 106(3), 804–812. 10.1152/japplphysiol.90872.2008 19131480

[jcp28923-bib-0051] Rullman, E. , Rundqvist, H. , Wagsater, D. , Fischer, H. , Eriksson, P. , Sundberg, C. J. , & Gustafsson, T. (2007). A single bout of exercise activates matrix metalloproteinase in human skeletal muscle. Journal of Applied Physiology, 102(6), 2346–2351. 10.1152/japplphysiol.00822.2006 17255365

[jcp28923-bib-0052] Sandri, M. (2008). Signaling in muscle atrophy and hypertrophy. Physiology, 23(3), 160–170. 10.1152/physiol.00041.2007 18556469

[jcp28923-bib-0053] Sasai, N. , Agata, N. , Inoue‐Miyazu, M. , Kawakami, K. , Kobayashi, K. , Sokabe, M. , & Hayakawa, K. (2010). Involvement of PI3K/Akt/TOR pathway in stretch‐induced hypertrophy of myotubes. Muscle and Nerve, 41(1), 100–106. 10.1002/mus.21473 19768770

[jcp28923-bib-0054] Schmittgen, T. D. , & Livak, K. J. (2008). Analyzing real‐time PCR data by the comparative CT method. Nature Protocols, 3(6), 1101–1108. 10.1038/nprot.2008.73 18546601

[jcp28923-bib-0055] Shansky, J. , Del Tatto, M. , Chromiak, J. , & Vandenburgh, H. (1997). A simplified method for tissue engineering skeletal muscle organoids in vitro. In Vitro Cellular & Developmental Biology. Animal, 33(9), 659–661. 10.1007/s11626-997-0118-y 9358276

[jcp28923-bib-0056] Shibahashi, K. , Sugiyama, K. , Kashiura, M. , & Hamabe, Y. (2017). Decreasing skeletal muscle as a risk factor for mortality in elderly patients with sepsis: A retrospective cohort study. Journal of Intensive Care, 5(1), 8 10.1186/s40560-016-0205-9 28096999 PMC5225584

[jcp28923-bib-0057] Shimizu, K. , Genma, R. , Gotou, Y. , Nagasaka, S. , & Honda, H. (2017). Three‐dimensional culture model of skeletal muscle tissue with atrophy induced by dexamethasone. Bioengineering (Basel, Switzerland), 4(2), 1–11. 10.3390/bioengineering4020056 PMC5590463 28952535

[jcp28923-bib-0058] Smith, A. S. T. , Passey, S. , Greensmith, L. , Mudera, V. , & Lewis, M. P. (2012). Characterization and optimization of a simple, repeatable system for the long term in vitro culture of aligned myotubes in 3D. Journal of Cellular Biochemistry, 113(3), 1044–1053. 10.1002/jcb.23437 22065378

[jcp28923-bib-0059] Spangenburg, E. E. , Le Roith, D. , Ward, C. W. , & Bodine, S. C. (2008). A functional insulin‐like growth factor receptor is not necessary for load‐induced skeletal muscle hypertrophy. The Journal of Physiology, 586(1), 283–291. 10.1113/jphysiol.2007.141507 17974583 PMC2375552

[jcp28923-bib-0060] Vandenburgh, H. , & Kaufman, S. (1979). In vitro model for stretch‐induced hypertrophy of skeletal muscle. Science, 203(4377), 265–268. 10.1126/science.569901 569901

[jcp28923-bib-0061] Vandenburgh, H. H. , Hatfaludy, S. , Karlisch, P. , & Shansky, J. (1989). Skeletal muscle growth is stimulated by intermittent stretch‐relaxation in tissue culture. American Journal of Physiology, 256(3 Pt 1), C674–C682. http://www.ncbi.nlm.nih.gov/pubmed/2923199 2923199 10.1152/ajpcell.1989.256.3.C674

[jcp28923-bib-0062] Velloso, C. P. (2008). Regulation of muscle mass by growth hormone and IGF‐I. (March), 557–568. Retrieved from https://saunaspace.com/wp‐content/uploads/2015/07/Regulation‐of‐muscle‐mass‐by‐growth‐hormone‐and‐IGF‐I.pdf 10.1038/bjp.2008.153 PMC2439518 18500379

[jcp28923-bib-0063] Wullschleger, S. , Loewith, R. , & Hall, M. N. (2006). TOR signaling in growth and metabolism. Cell, 124(3), 471–484. 10.1016/j.cell.2006.01.016 16469695

[jcp28923-bib-0064] Yang, Y. , Jemiolo, B. , Trappe, S. , & Proteolytic, S. T. (2006). Proteolytic mRNA expression in response to acute resistance exercise in human single skeletal muscle fibers. Journal of Applied Physiology, 101, 1442–1450. 10.1152/japplphysiol.00438.2006 16840578

